# Eye see through you! Eye tracking unmasks concealed face recognition despite countermeasures

**DOI:** 10.1186/s41235-019-0169-0

**Published:** 2019-08-07

**Authors:** Ailsa E. Millen, Peter J. B. Hancock

**Affiliations:** Psychology, Faculty of Natural Sciences, University of Stirling, Stirling, UK

**Keywords:** Markers of recognition, Familiar face recognition, Concealed Information Test, Countermeasures, Eye movement strategies

## Abstract

**Background:**

Criminal associates such as terrorist members are likely to deny knowing members of their network when questioned by police. Eye tracking research suggests that lies about familiar faces can be detected by distinct markers of recognition (e.g. fewer fixations and longer fixation durations) across multiple eye fixation parameters. However, the effect of explicit eye movement strategies to concealed recognition on such markers has not been examined. Our aim was to assess the impact of fixed-sequence eye movement strategies (across the forehead, ears, eyes, nose, mouth and chin) on markers of familiar face recognition. Participants were assigned to one of two groups: a standard guilty group who were simply instructed to conceal knowledge but with no specific instructions on how to do so; and a countermeasures group who were instructed to look at every familiar and unfamiliar face in the same way by executing a consistent sequence of fixations.

**Results:**

In the standard guilty group, lies about recognition of familiar faces showed longer average fixation durations, a lower proportion of fixations to the inner face regions, and proportionately more viewing of the eyes than honest responses to genuinely unknown faces. In the countermeasures condition, familiar face recognition was detected by longer fixations durations, fewer fixations to the inner regions of the face, and fewer interest areas of the face viewed. Longer fixation durations were a consistent marker of recognition across both conditions for most participants; differences were detectable from the first fixation.

**Conclusion:**

The results suggest that individuals can exert a degree of executive control over fixation patterns but that: the eyes are particularly attention-grabbing for familiar faces; the more viewers look around the face, the more they give themselves away; and attempts to deploy the same fixation patterns to familiar and unfamiliar faces were unsuccessful. The results suggest that the best strategy for concealing recognition might be to keep the eyes fixated in the centre of the screen but, even then, recognition is apparent in longer fixation durations. We discuss potential optimal conditions for detecting concealed knowledge of faces.

**Electronic supplementary material:**

The online version of this article (10.1186/s41235-019-0169-0) contains supplementary material, which is available to authorized users.

## Significance

Suppose you are being questioned by police about a crime. They show you a picture of the victim, or of your accomplice, either of whom you should know only if you were involved. You therefore deny knowing them. Our aim was to detect recognition of faces, not lies more generally.

The Concealed Information Test (CIT) is used in field practice to uncover guilty knowledge about a crime—such as a murder weapon, which only the person who did it should know. Used carefully, the CIT is a useful source of information, unlike standard lie-detector tests which are error-prone. There is, however, little work on using the CIT with faces. Our experiment is the first to examine the robustness of recognition markers in eye fixations during spontaneous and informed countermeasures to conceal recognition of faces with a sequential CIT.

We found that when liars spontaneously limited eye movements to conceal knowledge of faces, markers of recognition persisted in longer fixations and disproportionate viewing of the eyes and inner face regions. When liars attempted to execute fixed-sequence eye movements to conceal knowledge, they averted gaze from the eyes, but recognition was detected by differences in fixation durations, the number of face areas viewed and the proportion of fixations to inner face regions. In sum, liars were not able to fully counter markers of recognition either spontaneously or via informed countermeasures. These results suggest that it is difficult to conceal multiple markers of recognition simultaneously. Optimal procedures for the eye movement CIT are yet to be established.

## Background

Police officers routinely use photographs of faces to establish key identities in crimes. Some witnesses are honest, but many are hostile and intentionally conceal knowledge of known identities. For example, criminal networks, such as terrorist groups, will deny knowledge to protect one another. A victim might also be too afraid to identify their attacker. The liar’s goal is to hide any cues that might reveal their knowledge; the officer’s job is to look beyond the verbal denial and establish the truth.

Our aim was to use eye tracking to detect such deception, not by looking for signs of lying directly, but by looking for signs of recognition. Our approach combined two distinct strands of research: the applied detection of recognition through the Concealed Information Test (CIT); and theoretical models of familiar and unfamiliar face recognition. Critically, the current research assessed the vulnerability of markers of recognition (e.g. fewer, longer fixations) under explicit countermeasure instructions to look at both familiar and unfamiliar faces in the same way (CMs). To our knowledge, this is the first study to directly examine the effect of fixation-based countermeasure strategies on detection of familiar face recognition with a standardised sequential CIT.

## The Concealed Information Test

The CIT is the most scientifically validated protocol for the detection of concealed knowledge (for reviews, see Ben-Shakhar & Elaad, 2003; Meijer, Selle, Elber, & Ben-Shakhar, 2014; Suchotzki, Verschuere, Van Bockstaele, Ben-Shakhar, & Crombez, 2017; Verschuere, Ben-Shakhar, & Meijer, 2011). The success of the CIT is based on its solid theoretical foundation, resulting in high hit rates and exceptionally low false incrimination of innocents (0% in one field report; Hira & Furumitsu, 2002). The essence of the CIT is to present one critical item (probe), such as the murder weapon, amongst many irrelevant items of the same type. For example, if the murder weapon was a gun, it might be presented in a sequence of items including a knife, rope, hammer or axe. A genuinely innocent person has no reason to respond differently to any of the items since none should be familiar or meaningful. For the guilty suspect, however, the critical item produces a reflexive orienting response (OR) enhanced by its rare presentation amongst many irrelevant items (Lykken, 1959, 1960; Sokolov, 1963, 1966). In Japan, approximately 5000 CITs are conducted each year using autonomic measures of recognition (e.g. skin conductance) based on knowledge of crime details. However, to date there has been little work evaluating the CIT for detecting recognition of faces.

Successful attempts to detect concealed face recognition in the laboratory have been achieved by combining the CIT with fMRI (Bhatt et al., 2009) and P300-based ERP approaches (e.g., Meijer, Smulders, Merckelbach, & Wolf, 2007; Meijer, Smulders, & Wolf, 2009). However, both are invasive, and in the case of fMRI likely always to be prohibitively expensive. In addition, both ERP and fMRI CITs are also known to be susceptible to simple physical countermeasures such as wiggling a finger or toe in response to selected irrelevant items (e.g., Ganis, Rosenfeld, Meixner, Ra, & Schendan, 2011; Rosenfeld, Soskins, Bosh, & Ryan, 2004). Attempts to detect concealed face recognition with simpler reaction time-based CITs (RT-CITs) are mixed. Seymour, Baker, and Gaunt (2013) achieved 98% classification accuracy for detecting familiar face recognition via longer RTs. However, the combination of an elaborate study phase before the CIT and use of the same images at study and at test limits the meaningfulness of the finding. Simple image recognition is rather elementary and does not represent face recognition as it occurs in real life. Using the same image at study and at test is likely to inflate detection rates since individuals are only required to match an image recently stored in memory, as opposed to recalling a known identity from long-term memory. This point is nicely illustrated by Georgiou, Chronos, Verschuere, and Sauerland (2019), who studied factors affecting the detection of recognition of a face seen in a mock crime video. Showing the same photograph to be used at test after the video increased the effect size from *d* = 0.64 to *d* = 1.21. However, another series of experiments by the same research group using mock crime videos and a virtual reality paradigm were unable to detect concealed recognition of newly familiar faces that were viewed briefly before the test (*d* = 0.14; Sauerland, Wolfs, Crans, & Verschuere, 2017). Furthermore, some researchers have expressed concerns about the vulnerability of the simple RT-CIT to simple countermeasures (Farwell & Donchin, 1991; Gronau, Ben-Shakhar, & Cohen, 2005; Varga, Visu-Petra, Miclea, & Bus, 2014). Systematic research on the vulnerability of the RT-CIT to countermeasures is scarce; no studies have examined this factor in relation to faces.

## Using eye tracking to detect familiar face recognition

Eye tracking technology shows clear potential for use with the CIT. Distinct differences in the processing of familiar and unfamiliar faces are extensively documented in basic eye movement research (for an extensive review, see Hannula et al., 2010). In sequential face recognition tasks, familiar faces elicit fewer fixations, fewer areas of the face viewed (e.g. eyes, nose, face and mouth), fewer return fixations to previously viewed areas of interest, smaller proportions of fixations to the inner regions of the face (Althoff & Cohen, 1999) and longer fixation durations (Ryan, Hannula, & Cohen, 2007; Schwedes & Wentura, 2012); herein referred to as markers of recognition. These findings reflect the relative ease with which familiar faces are recognised, compared to unfamiliar faces (Balas, Cox, & Conwell, 2007; Hancock, Bruce, & Burton, 2000; Johnston & Edmonds, 2009; Natu & O’Toole, 2011; Ramon & Gobbini, 2018), consistent with theoretical models of face perception which document that recognition of a familiar face is rather more holistic than the identification of genuinely unfamiliar faces, which tends to rely more on part-based featural information extraction (Bruce & Young, 1986; Burton, Bruce, & Hancock, 1999; Burton, Jenkins, & Schweinberger, 2011; Collishaw & Hole, 2000; Gobbini et al., 2013; Gobbini & Haxby, 2007; Schacter, Norman, & Koutstaal, 1998).

Crucially, it has been suggested that markers of recognition during familiar face viewing may be obligatory, or even involuntary (Hannula et al., 2010; Ryan et al., 2007). For example, Bate, Haslam, Tree, and Hodgson (2008) found markers of recognition in a sample of participants with prosopagnosia (i.e. face blindness). Others found similar results under instructions to avoid looking at the face (Ryan et al., 2007) or when instructed to look at each familiar and unfamiliar face in the same face way (moving from the forehead to the eyes and ears, then nose mouth and chin; Althoff & Cohen, 1999). This reported feature of eye fixations makes them a strong candidate for the detecting of concealed recognition during lies but, as yet, the ability to control eye fixations during recognition and explicit denial of knowledge in response to singly presented faces is untested.

In studies of concealed face recognition, Millen, Hope, Hillstrom, and Vrij (2017) found that the same markers of recognition (e.g. fewer total fixations on the face, fewer regions of face viewed) signalled face familiarity during lies about well-known faces, including personally known faces (*d* = 0.9) and famous celebrities (*d* = 0.5), compared with unknown faces. Faces only seen briefly before the experiment, however, produced less clear differences in fixation patterns (*d* = 0.2). These results led the authors to suggest that personal familiarity may be particularly robust to deception due to the reflexive nature of recognition for well-known faces. However, only 32% of liars in Millen et al.’s’ study attempted to deploy any sort of deliberate countermeasure. Furthermore, the study did not assess the vulnerability of recognition markers (e.g. fewer, longer fixations) under explicit countermeasure instructions to control eye fixations to conceal recognition. Accordingly, the conclusion that markers of recognition for personal familiarity are robust to intentional deception warrants further attention. In other studies, longer fixation durations indexed lies about recognition within 250–500 ms during a face–scene associative memory task (Mahoney, Kapur, Osmon, & Hannula, 2018) and the second fixation during lies in response to six-face displays comprising one familiar face presented among five unfamiliar faces (Schwedes & Wentura, 2012; see also Schwedes & Wentura, 2016). The early onset of increased fixation durations during recognition suggests that such markers of recognition may be particularly difficult to control; a promising feature for potential markers of recognition during deceit. A notable feature of fixation durations is that they generally increase with additional cognitive load (Castelhano & Rayner, 2008; Rayner, 1998). In the current experiment, we expect that the average fixation durations will increase because of recognition orienting effects, in addition to cognitive load during lies (e.g. response conflict and strategies to conceal knowledge; Cook et al., 2012). The prediction is that the harder liars try to conceal knowledge, the easier it should be to detect.

Only one previous study has examined the effect of explicit countermeasure instructions to control eye movements during a CIT. Lancry-Dayan, Nahari, Ben-Shakhar, and Pertzov (2018) instructed participants to deny recognition of the familiar face whilst trying to direct their gaze equally to all faces on the screen to conceal recognition in a modified CIT with simultaneous presentation of four faces (one familiar and three unfamiliar). They found that the countermeasures instruction attenuated the initial orienting of gaze to the familiar face, but that concealed recognition was detected by an overt avoidance of the familiar face which differed to gaze patterns for trials that did not contain any unfamiliar faces at all. Lancry-Dayan et al.’s (2018) study offers a first insight into deceivers’ abilities to directly control eye fixations during concealed recognition. In another study, Peth, Suchotzki, and Gamer (2016) investigated the effect of physical (finger wiggling) and mental (imagining an emotional event) countermeasures to selected items whilst recording eye fixations and autonomic responses (e.g. skin conductance) during recognition of photographs of items from a mock crime. The main finding for their fixation data was that recognition was detected by differences in number of fixations in all three condition groups (standard guilty, physical countermeasures and mental countermeasures), but that fixation durations were longer only during concealed knowledge in the standard guilty group. Like Peth et al., we adopt the sequential CIT format but instead examine the robustness of fixation measures to explicit instructions to control eye movements using a fixed sequence viewing strategy for all faces. To our knowledge, there are currently no published studies that examine the extent to which intentional efforts to control fixations impact markers of recognition for presentation of single faces in a standardised CIT. It is reasonable to predict that recognition might be particularly strong for personally familiar faces and more difficult to conceal than newly familiar details of a mock crime (e.g., Peth, Kim, & Gamer, 2013).

Our goal was to conduct such a study with explicit countermeasure instructions to look the same way at each familiar and unfamiliar face. We utilise a standard sequential CIT format because it has been validated by much current research and field practice. If CIT examiners are to extend the use of the standard CIT to include faces, then it is crucial to examine the reliability of markers of recognition and their robustness to countermeasures. We examined markers of recognition in two critical conditions: a standard guilty condition where liars spontaneously attempted to conceal recognition of familiar faces without any information on how to do so (SG: Experiment 1a); and an informed countermeasure condition where liars were instructed to conceal recognition by moving their eyes in the same fixed sequence pattern across the forehead, ears, eyes, nose, mouth and chin (CM: Experiment 1b). With this design, we aimed to establish whether large effect sizes in markers of recognition for personally familiar faces reported by Millen et al. (2017) are robust during explicit countermeasures to look the same way at every face. Here, we adopt the same instructions as Althoff and Cohen (1999), who reported that the proportion of fixations to the inner regions of the face (eyes, nose and mouth) indicated memory despite attempts to look the same way at all faces. We predicted that markers of recognition (fewer fixations, fewer interested areas viewed, lower proportion of fixations to inner regions of the face) would more consistently detect recognition in the standard guilty condition than the countermeasures condition, but that longer fixation durations would detect recognition in both conditions.

## Methods

### Power analysis

A power analysis was calculated using G*Power (Faul, Erdfelder, Lang, & Buchner, 2007) with *d* = 0.7, an α error probability of 0.05 and a *β* value of 0.95, which estimated 24 participants in each group (*N* = 48). The predicted effect size for the current study is conservative compared to a similar study by Millen et al. (2017), who reported large effect sizes for detection of *personally familiar faces* by number of fixations (*d* = 0.9), number of areas of the face viewed (*d* = 0.9) and average fixation duration (*d* = 0.9). It also represents the mean effect size reported by Peth et al. (2013), *d* = 0.82 and *d* = 0.62 for number of fixations and fixation duration, respectively.

### Participants

Forty-eight undergraduate psychology students participated in the current experiment. In the standard guilty condition, participant ages ranged from 17 to 55 years (*M* = 24.9, *SD* = 9.49; 17 females, 7 males). In the countermeasures condition, participant ages ranged from 18 to 53 years (*M* = 23.5, *SD* = 9.03; 17 females, 7 males). Participants were recruited via the online psychology sign-up system or by email and social media. Our inclusion criteria were that participants had normal or corrected-to-normal vision and that they were familiar with the identities of lecturers presented in the study for at least one semester. Ethical approval was granted by the General University Ethics Panel at the University of Stirling (GUEP219).

### Design

In each CIT block, participants were shown a sequence of familiar and unfamiliar faces on a display screen. Within subjects, all participants made yes/no responses to faces (whilst verbalising their response) according to three basic task instructions: deny knowledge of one familiar face (no to familiar probe); correctly reject unfamiliar faces as unknown (no to unfamiliar irrelevants); and honestly identify a second familiar face (yes to familiar target), consistent with the Concealed Information Test Three-Stimulus Protocol (CIT-3SP; e.g., Sauerland, Wolfs, Crans, & Verschuere, 2017). Between subjects, half of the participants (*N* = 24) completed the task according to the basic standard guilty instructions to appear honest in all trials (SG), and half completed the task while trying to execute the fixed sequence of eye movements (CM). All participants completed four CITs, each with a new set of familiar and unfamiliar photographs. Yes/no responses were made via keyboard presses [z/m] concurrently with a verbal yes/no response. The CIT presentation order was randomised between participants in addition to the presentation of single faces within blocks. The [z] and [m] keys were counterbalanced for yes/no responses by handedness.

### Materials

#### Faces

Photographs of faces were full colour images with a neutral expression and gaze towards the camera. Adobe Photoshop CC was used to extract images from their original background, superimpose them on a standard black background and remove any defining features such as moles and jewellery. The IRFAN view was used to resize all images to 595 × 420 pixels (visual angles of 11.3° for height and 15.9° for width). Photographs of familiar and unfamiliar faces were sourced from the Psychological Image Collection at Stirling (PICS; http://pics.stir.ac.uk). The familiar faces were eight male psychology lecturers which participants had met repeatedly during their studies for at least one semester. In total, 72 unique images were shown over four blocks of trials (18 in each block). In each block, the 18 test images comprised 12 unfamiliar irrelevant faces, three different images of one lecturer (probes) and three different images of a second lecturer (target). We selected a different probe identity for each block consistent with the field CIT, which typically presents one item per question and repeats all questions three to five times. Instead of using the same images, we repeated the same identity within one CIT test block, using three different images of the same person. Twelve different unfamiliar faces were presented in each test block since repeating the same four images, as would typically be done in field practice, would likely result in familiarisation of the unfamiliar faces and attenuation of the probe-irrelevant CIT effect. Consistent with fair line-up procedures (Wells et al., 1998), unfamiliar faces in each block were selected for similarity to the familiar probe face based on a MATLAB face matching algorithm.^1^

#### Videos

Additionally, eight 5-s video clips of each lecturer speaking (sound muted) were created using Windows Live Movie Maker (version 2011, Build 15.4.3538.0513) and shown to participants prior to the CIT to check familiarity with each lecturer. Familiarity ratings were recorded as inclusion criteria for participation and are not analysed further here.

#### Questionnaire

Seven-point scales were used to assess self-reports of participants’ motivation to conceal recognition of designated faces during the test (1 = not motivated at all, 7 = very motivated), effort invested in the concealment of recognition (1 = no effort at all, 7 = great effort) and confidence that they successfully concealed recognition as instructed (1 = not very confident, 7 = very confident). One final open question asked participants to report any strategies they used to try and conceal recognition.

#### Apparatus

The experiment was programmed in E-prime (version 2.0.10.356) and integrated with SensoMotoric Instruments non-invasive image-based tracker (SMI RED250) via the E-prime extension 1.0. The sampling rate was set to 60 Hz and eyes were tracked via the pupil and corneal reflection. The experiment was executed via a laptop (model: DELL Latitude E6250) with a refresh rate of 60 Hz. The display screen was a 22-inch DELL monitor (model P2210) with a resolution of 1440 × 900 pixels. Raw data were managed with the SMI BeGaze software (version 3.6) dispersion-based algorithm. According to this algorithm, two main parameters are used to calculate fixations: a minimum fixation duration threshold, set here to 80 ms; and the maximum dispersion set to 100 pixels. This algorithm is explained in the BeGaze manual (SMI 2012) and matches the Dispersion Threshold Identification (I-DT) algorithm described by Salvucci and Goldberg (2000).

### Procedure

Prior to the CIT, we recorded whether participants were familiar with each of the eight lecturer identities to be shown across the four CITs. Each participant was shown a short video of each lecturer and asked to rate how familiar they were with that person on a 7-point scale (1 = not familiar at all, 7 = very familiar). Participants proceeded to the experiment unless they recorded being ‘not at all’ familiar with two or more of the probe faces to be shown in the experiment. During the experiment, participants were seated in a testing cubicle with fixed lighting conditions at 70 cm from the screen. The eye tracker was mounted on a vertically adjustable platform so that the screen was positioned centrally and participants’ eyes rested in the centre of the screen. Participants were instructed to keep as still as possible during the test trials, so that eye movements could be recorded freely without a chin rest. Basic information on participant details was recorded (age, gender, handedness and button assigned to familiar ‘yes’ response), followed by an instruction screen indicating which face they should deny knowledge of (say ‘no’ to probe) and which face should be honestly identified (say ‘yes’ to target). They were further instructed to respond honestly to all genuinely unfamiliar faces (say ‘no’ to irrelevants). Between-subjects’ groups received different task instructions. In the SG condition, participants were given the key instruction that they should conceal knowledge whilst attempting to appear honest during all trials. In the countermeasures condition (CM) they were additionally instructed to execute a fixed sequence of fixations to the face (forehead, right ear (left visual space), right eye (left visual space), left eye (right visual space), left ear (right visual space), nose, mouth and chin).

**Fig. 1:**
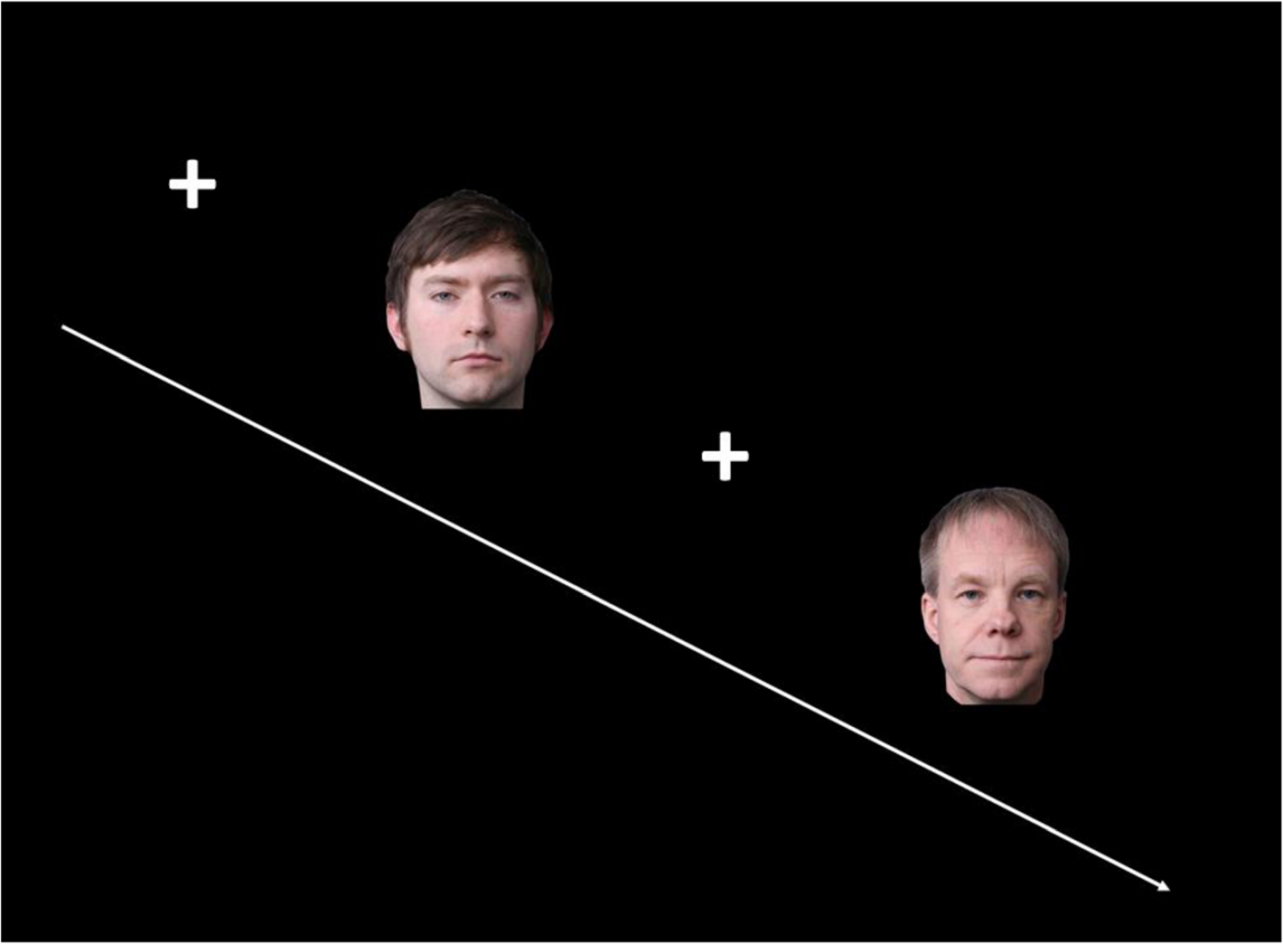
Trial sequence diagram

Task instructions were followed by a 9-point calibration and CIT trials commenced following accuracy within 0.4° (spatial resolution 0.03°). New test instructions for each new identity set, and additional calibrations, were conducted prior to each test block. The trial sequence within each block commenced with presentation of two unfamiliar faces as practice trials (buffers) followed by the remaining 18 faces (12 irrelevants, 3 probes, 3 targets) presented in a random sequence. Participants made yes/no responses to each face in turn (no to the three different images of the same probe, yes to the three different images of the same target and no to each unfamiliar irrelevant face), whilst at the same time stating their yes/no response out loud (see Fig. 1). There was no upper time limit for the response. The E-prime script recorded behavioural data including experiment trial variables. The iViewX operating system for the RED250 recorded ocular parameters at a rate of 60 measures per second (60 Hz).

### Data analysis

#### Markers of recognition

We selected four markers of recognition based on their theoretical relevance. The number of fixations indicates a marker of general cognitive effort (Num. Fixations). The number of different interest areas of the face viewed (IAs Visited; left eye, right eye, nose, mouth, outer) reflect the amount of information required for recognition. The proportion of fixations made to the inner regions of the face including the eyes, nose and mouth (Proportion Inner) reflects the extent to which recognition is achieved from inspecting critical inner face features. Finally, the average fixation duration (AFD) was recorded as an index of depth of processing. The AFD was calculated by summing the length of all fixations made to the item and then dividing by the number of total fixations.

#### Cohen’s *d* effect size analyses

The detection of concealed knowledge is concerned with identifying robust markers of recognition via large effect sizes with narrow confidence intervals. Accordingly, we present our data across both experiments as Cohen’s *d* effect size differences with 95% confidence intervals (CIs) for concealed knowledge of familiar items (familiar probes) compared to correct rejections of genuinely unknown items (unfamiliar irrelevants). Here, Cohen’s *d* is calculated based on standardised difference scores for each dependent variable (e.g., Ben-Shakhar, 1985).

For each participant, three random irrelevant items were first removed from each block equal to the number of valid probe responses to allow simulation of a virtual ‘innocent’ group of responses for the receiver operating characteristic (ROC) analyses outlined in the following. The mean and standard deviation of the remaining irrelevant responses was then used to compute *z*-scores for the innocent and probe items. This process was repeated 1000 times to compute mean values for Cohen’s *d* and the ROC and area under the curve (AUC), with 95% CIs.

#### Receiver operating characteristic curves

To determine the detection efficiency of the markers of recognition we calculated ROC curves to plot the true positive rate (i.e. sensitivity) against the false positive rate, giving an AUC value for the detection rate. The AUC represents the efficacy of different recognition markers for differentiating concealed (familiar faces) from truthful (unfamiliar faces) responses. AUC values range from 0 to 1, with 0.5 indicating chance detection (see also Ben-Shakhar, Lieblich, & Kugelmass, 1970). To perform ROC classifications, an average *z*-score for innocent irrelevants and probe items was computed for each participant. MATLAB® was used to calculate Cohen’s *d* and the AUC with 95% bootstrapped CIs.

## Results

The raw data, standardised scores and resampling script are available on the open science framework (https://osf.io/k2aut/).

## Exclusions

One participant was excluded from the dataset for not performing the task according to instructions. This participant was replaced so that the total number of participants was consistent with the planned sample size (*N* = 48).

Individual trials were removed including incorrect yes responses to familiar faces during lie trials (23 out of 288) and incorrectly responding that an unfamiliar face was familiar (17 out of 1152). A total 1100 trials were analysed out of a possible 1140.

## Markers of recognition

**Fig. 2:**
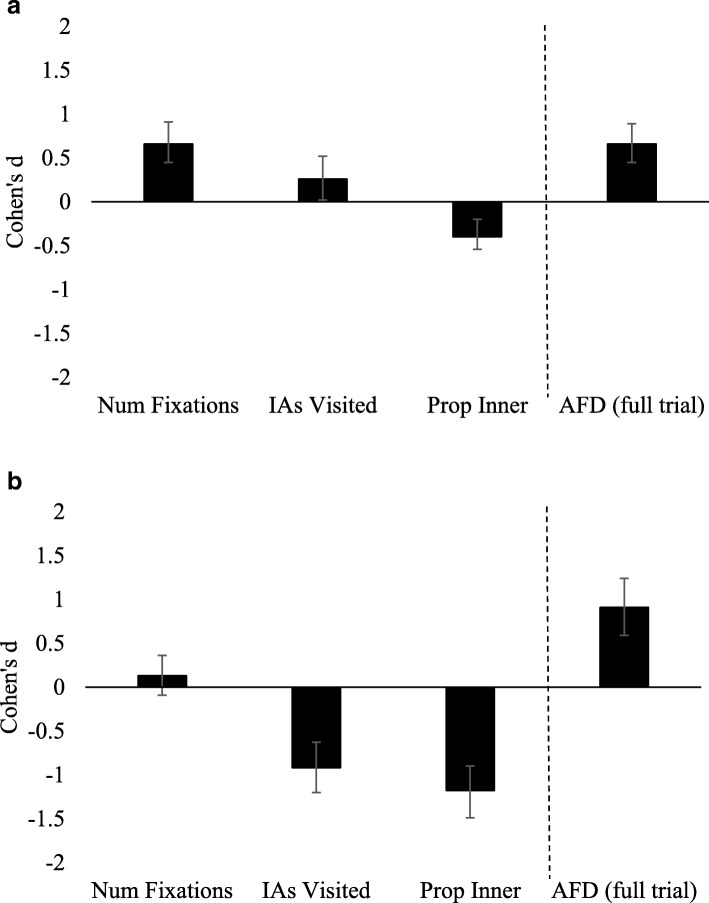
Cohen’s *d* effect sizes for each of the four markers of recognition: number of total fixations to the face (Num. Fixations), the number of different interest areas of the face viewed including left eye, right eye, nose, mouth and outer (IAs Visited), the proportion of all fixations made to the inner regions of the face (Prop. Inner) and the average fixation duration calculated by the sum of all fixations divided by the total number of fixations over the full trial (AFD full trial): **a** standard guilty condition; **b** countermeasures condition. Errors bars represent 95% confidence intervals on the Cohen’s *d* effect size

Figure 2a (standard guilty) and Figure 2b (countermeasures) show Cohen’s *d* effect sizes for each of the four selected fixation measures during concealed recognition of familiar faces, compared to correct rejection of genuinely unfamiliar faces (*M*_familiarProbe_ – *M*_unfamiliarIrrelevant_). In all cases, a positive *d* value indicates that the measure for probe items was higher than for innocents. For three of the measures, Num. Fixations, IAs Visited and Proportion Inner, the prediction is that scores should be lower during concealed recognition compared to honest responses to genuinely unfamiliar faces. The prediction is that average fixation durations should be longer for familiar items. The vertical dashed line indicates the point at which data consistent with predictions should change from negative to positive values

## Confirmatory analyses

### Results summary 1: markers of recognition

In the standard guilty condition (SG; Fig. 2a), where participants were simply instructed to conceal knowledge and appear honest in all trials, longer average fixation durations (AFDs) signalled recognition of familiar faces. In the countermeasures condition (CM; Fig. 2b), longer AFDs also signalled recognition. The effect size difference in AFDs between familiar and unfamiliar faces was larger in the CM condition (*d* = 0.91, 95% CI [0.59, 1.24]) than the SG condition (*d* = 0.66, 95% CI [0.45, 0.89]). Longer fixation durations distinguished familiar face recognition from rejection of genuinely unfamiliar faces as early as the first fixation in both the SG condition (*d* = 0.72, 95% CI [0.49, 0.96]) and the CM condition (*d* = 0.62, 95% CI [0.33, 0.87]).

In the SG condition familiar faces did not elicit fewer fixations or fewer IAs Visited, but the proportion of fixations to inner face regions was lower for familiar faces as predicted. In the CM condition, familiar faces elicited fewer IAs Visited and a lower proportion of fixations to the inner regions of the face, but there was no difference in total Num. Fixations.

Further inspection of the mean number of total fixations made to familiar and unfamiliar faces showed that, for both face types, barely more than one or two fixations were made before the response; familiar probes, *M* = 2.3, *SD* = 1.94; unfamiliar irrelevants, *M* = 1.95 *SD* = 1.18. The equivalent mean total fixations for the CM condition were: familiar probe faces, *M* = 9.82, *SD* = 4; unfamiliar irrelevant faces, *M* = 9.84, *SD* = 3.6. The results suggest that the general instruction to conceal recognition in the SG condition caused liars to control the number of fixations prior to the response. To examine whether any differences emerged in where liars looked during that time, we further examined the proportion of time spent looking at different areas of the face including the eyes (left and right combined), the nose, the mouth and the outer areas of the face (all parts of the face excluding the inner areas and including the forehead, ears, jaw, chin, etc). See Additional file 1: Figure S1 for a hot-spot illustration of differences in fixations patterns across standard guilty and countermeasure condition instructions for a familiar probe and one unfamiliar irrelevant face.

## Exploratory analyses

### Results summary 2: interest area analyses

**Fig. 3:**
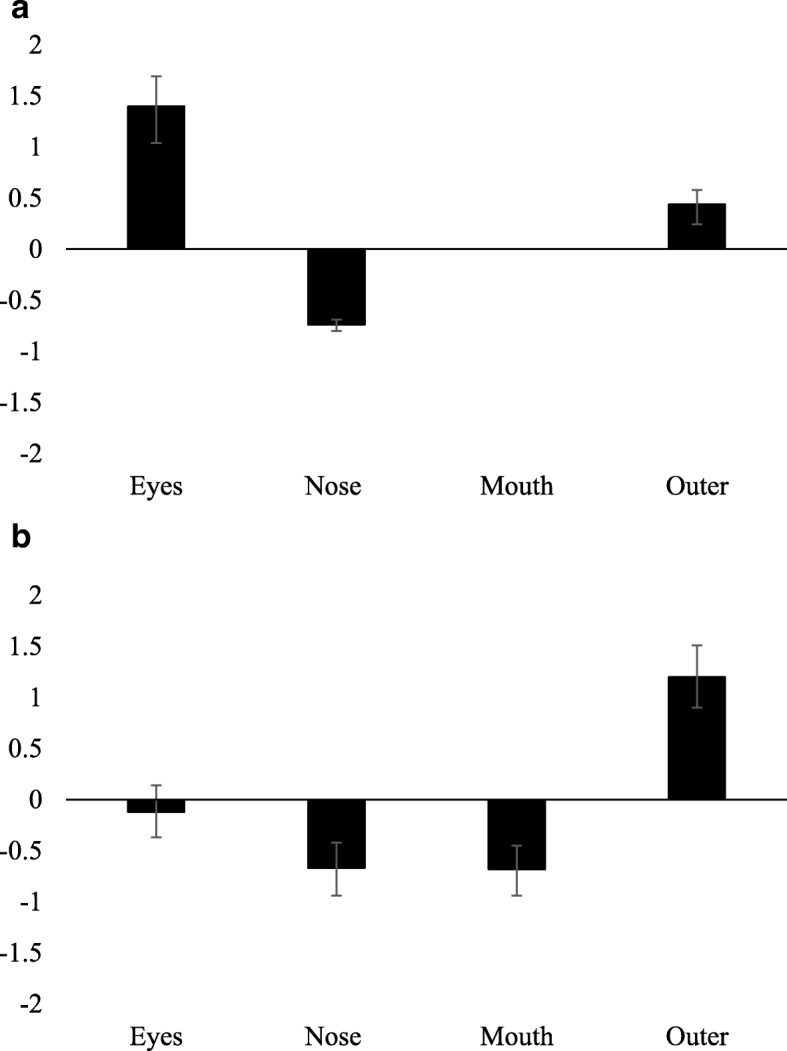
Differences in proportions of total fixations made to interest areas of the face. Differences in proportions of total fixations made to interest areas of the face between probe and irrelevant items: **a** standard guilty condition; **b** countermeasures condition. Note that no data are present for the ‘mouth’ interest area since, in this condition, there were only 13 instances of looking at the mouth out of 1397 correct trials. Thus, *z*-scores could not be meaningfully calculated. Error bars represent 95% CIs on Cohen’s *d* effect size

In the standard guilty condition (SG; Fig. 3a), despite making very few fixations, recognition of familiar faces produced a significantly higher proportion of fixations to the eye region, distinguishing lies about familiar face recognition from correct rejection of genuinely unfamiliar faces (*d* = 1.34, 95% CI [0.78, 1.89]). Participants also made fewer fixations to the nose during viewing of familiar faces, d = -0.74, 95% CIs [-0.80, -0.69], and almost never looked at the mouth. The effect of increased eye viewing disappeared in the countermeasures condition (CM; Fig. 3b) when liars executed a fixed-sequence eye movement strategy to conceal recognition. However, despite trying to look the same way at familiar and unfamiliar faces, a smaller proportion of fixations to the inner regions of the face, driven by decreased viewing of the nose and mouth, distinguished familiar face recognition in this condition.

## Receiver operator curve characteristic analyses

The AUC values (Table 1) confirmed the fixation duration (first AFD, AFD), proportion of fixations to the inner regions of the face, and proportion of fixations to the eyes as reliable markers of recognition in the standard guilty condition. In the countermeasures condition, IAs Visited, Proportion Inner and the average fixation duration (first AFD, AFD) detected concealed recognition over chance^2^.

**Table 1:** Area under the curve calculated from ROC analyses, with lower and upper 95% CIs

	Standard guilty			Countermeasures		
	*a*	Lower	Upper	*a*	Lower	Upper
Num. Fixations	0.34	0.27	0.41	0.47	0.40	0.54
IAs Visited	0.45	0.38	0.52	**0.76**	0.69	0.82
Proportion Inner	**0.61**	0.54	0.67	**0.80**	0.74	0.86
AFD	**0.67**	0.60	0.74	**0.74**	0.66	0.81
First AFD	**0.69**	0.62	0.77	**0.66**	0.59	0.73
Eyes	**0.87**	0.81	0.92	0.48	0.40	0.56

Numbers in bold indicate classification over chance. *AFD* average fixation duration, *CI* confidence interval, *IAs Visited* number of different interest areas of the face viewed, *Num. Fixations* number of total fixations to the face, *Proportion Inner* proportion of all fixations made to the inner regions of the face, *ROC* receiver operating characteristic

In sum, fixation duration and proportion of fixations to the inner regions of the face were consistent markers of recognition across participants and conditions. The AUC values in the table correspond to detection of recognition by longer average fixation duration (AFD) for 68.8% of participants in the standard guilty condition and 76.1% in the countermeasures condition (*z*-scores in predicted direction). Similar results were observed in the first fixation duration (66.7% of participants in the standard guilty condition and 67.5% in the countermeasures condition). The proportion of fixations to the inner face regions was lower during familiar face recognition for 57% of participants in the standard guilty and 83.5% participants in the countermeasures condition. See Additional file 1: Table S1 for a full report of measures as percentages.

## Discussion

We aimed to explore the robustness of recognition markers (fewer, longer fixations) to personally familiar faces during spontaneous attempts to conceal recognition and during explicit countermeasures instructions to look at familiar and unfamiliar faces with the same viewing pattern. First, we predicted that the standard guilty condition would replicate the same pattern of results as previous research for personally familiar faces, including fewer fixations, fewer interest areas viewed, lower proportion of fixations to the inner regions of the face (Millen et al., 2017) and longer fixation durations (Mahoney et al., 2018; Schwedes & Wentura, 2012). Second, we predicted that countermeasures strategies would obscure detection of recognition by fixation count, number of interest areas viewed and proportion of fixations to the inner regions of the face, but that longer fixation durations would be a reliable indicator of concealed knowledge across both conditions.

When participants were simply instructed to appear honest in all trials (standard guilty), recognised faces elicited longer fixation durations, a higher proportion of fixations to the eyes, and lower proportion of fixations directed to the combined inner regions of the face (eyes, nose and mouth) compared to unknown faces. When participants were instructed to look at every face the same way (countermeasures), recognition was signalled by longer fixation durations, fewer areas of the face viewed and lower proportion of fixations directed to the inner regions of the face. Our novel findings, which partially supported our predictions, present key insights into procedural considerations for the optimal elicitation of fixation markers during an eye movement-based CIT.

In the standard guilty condition, longer fixation durations signalled recognition of familiar faces in the first fixation (*a* = 0.69) and total average fixation duration (*a* = 0.67). Our pattern of results is somewhat consistent with previous research using multiple face displays formats to detect recognition of familiar faces. Schwedes and Wentura (2012) found longer fixations in the second fixation with 65% detection accuracy (total fixation durations), whereas Lancry-Dayan et al. (2018) combined two opposing gaze patterns to detect concealed recognition (early orienting to the familiar faces followed by gaze aversion) and achieved *a* = 0.89 classification accuracy. More generally, our finding is also consistent with Peth et al. (2013, 2016), who found that longer fixation durations signalled concealed recognition of non-face objects central to a mock crime scenario (*a* = 0.69 and *a* = 0.73) during a sequential CIT.

Our findings did not replicate previous research using standard guilty instructions with regards to fewer fixations and, fewer interest areas viewed (e.g., Millen et al., 2017). The findings are also not consistent with the work by Peth et al. (2013), who reported fewer fixations during recognition of crime details that were central to a mock crime (*a* = 0.82), compared to irrelevant items. In a later study, they also found that recognition was indicated by fewer fixations even when participants executed physical (finger wiggling) and mental (imagining an emotional event) countermeasures to selected irrelevant items (Peth et al., 2016). In the current results, inspection of mean fixation counts revealed that individuals made only one or two fixations before responding to faces, which limited scope for detection via the quantity and distribution of fixations. The finding that familiar faces were recognised in approximately two fixations is consistent with previous research which has reported that two fixations are sufficient for accurate familiar face identification (Hsiao & Cottrell, 2008). This result is also consistent with reports of fast recognition for familiar faces and theoretical accounts of familiar and unfamiliar face processing (Hancock et al., 2000; Johnston & Edmonds, 2009).

Unexpected results, however, were found in relation to correct rejection of genuinely unfamiliar faces. Responses to unfamiliar faces were made, on average, in fewer than two fixations. Our results suggest that individuals did not approach the current concealed face recognition test as they might do in typical recognition experiments, or indeed in real-life encounters, where each response depends on careful consideration of multiple possible identities. In contrast to our own experimental design, Millen et al.’s (2017) experiment employed a design with multiple and equal numbers of unfamiliar, newly learned and personally familiar faces in each test block. The complexity of their task demanded that attention was paid to each face to adhere to instructions that inconsistent errors would signal suspicion. Millen et al.’s design more closely represents the complexity of identity evaluations in the real world, where familiarity with different people varies by group (e.g. friend vs acquaintance) and degree (well known vs newly familiar). In the current experiment, however, we employed the standard CIT paradigm which presented only one personally familiar probe identity (deny knowledge) and one personally familiar target (honestly identify) amongst many unfamiliar irrelevant items in each test block.

Results in the standard guilty condition suggest that a single-probe CIT, with a single probe identity per block, may not be best suited to the capture of eye movement patterns. However, there is no evidence to suggest that this format was detrimental to the detection of recognition by fixation duration. We observed that liars were able to make fast responses to unfamiliar faces within limited fixations. The inclusion of the target item (a familiar face for which responses should be truthful) to maintain attention and to limit systematic responding and countermeasures efforts was less effective than intended. For example, our data suggest that individuals were able to respond no to the question ‘Is it familiar probe “X”?’ or ‘Is it unfamiliar irrelevant “Y”?’ with minimal fixations. With this approach it appears that individuals were able to quickly identify whether each face was probe ‘x’ or target ‘z’, and rapidly reject unfamiliar irrelevant faces as not being the probe/target without having to fully engage in more elaborate processes of recollection or familiarity beyond the two personally familiar identities directly relevant to the task (Jacoby, 1991; Jacoby, Toth, & Yonelinas, 1993; Yonelinas, 2002). We suggest that knowing the identity of the person for whom they would deny knowledge before the test made this task particularly easy, in addition to both probe and target identities being personally familiar. The use of a newly familiar target (previously unknown) might minimise such rapid exclusion of probes and targets in future studies (see Georgiadou, Chronos, Verschuere, & Sauerland, 2019, and Sauerland, Wolfs, Crans, & Verschuere, 2017, who use newly familiar targets and more than one probe in each CIT test block). Whether this would apply to real-world usage would depend on the protocol being used. If the suspect is not forewarned of which faces may be shown, the strategy cannot be used. However, standard protocol in Japan is to display all items before the test begins to familiarise the interviewee with test items and to remove any items that stand out. There is no evidence that previewing test items hinders detection with the autonomic CIT (Verschuere & Crombez, 2008), but the impact of previewing items on alternative CIT methods such as eye movement-based protocols is yet to be tested.

The countermeasure instructions were successful in obscuring some differences between familiar probes and unfamiliar irrelevant faces. For example, fixation time on the eyes gave an effect size of *d* = 1.4 in the SG free viewing which disappeared completely in the CM group (*d* = − 0.12). On the other hand, the CM group showed an increased effect size for some measures such as time spent on inner features (*d* = − 0.4 to − 1.2), which grab attention differently for familiar and unfamiliar faces despite the attempt to execute a fixed pattern of movements. The finding that a lower proportion of fixations was made to the inner face regions of familiar faces during both the standard guilty and countermeasures conditions is consistent with previous work by Althoff and colleagues (1999) who reported that participants made fewer fixations to inner face regions during both free viewing of faces and during explicit instructions to view familiar and unfamiliar faces using a fixed sequence viewing pattern (across the forehead, ears, eyes, nose, mouth and chin). Fixation duration effect size also increased in the CM group (*d* = 0.7 to *d* = 0.9) with differences in the first fixation. Fixation duration results support the finding that effects of recognition are fast and reflexive (e.g., Gobbini et al., 2013) and that both recognition and effort to conceal recognition (e.g. response conflict and countermeasure strategies) contribute to longer fixation durations (e.g., Cook et al., 2012).

The optimal format for eye movement-based CITs is unknown and substantially more research is required to clarify optimal methodological procedures in this field. The most appropriate test format for each CIT must depend on the variable and mechanism of interest. For example, increasing mental workload and task demands are favourable for reaction time-based CITs, which rely on longer response times to detect recognition (Suchotzki et al., 2017; Verschuere, Kleinberg, & Theocharidou, 2015). Accordingly, RT-CIT researchers tend to utilise multiple-probe CITs (multiple familiar probe items presented within one block) for optimal detection of crime details (e.g., Sauerland, Wolfs, Crans, & Verschuere, 2017; Seymour & Kerlin, 2008; Verschuere, Crombez, Degrootte, & Rosseel, 2010). Conversely, CIT researchers utilising event-related brain potential or physiological measures to detect recognition based on an orienting response favour the single-probe CIT block and multiple repetitions of items (Ben-Shakhar & Elaad, 2003, Rosenfeld, Shue, & Singer, 2007. The current results, combined with Millen et al.’s (2017) previous findings, suggest that including more than one probe in each CIT and including a less familiar target might be better for detecting recognition of well-known faces during attempts to constrain fixations. However, in real life there might not be more than one key identity for which investigators wish to probe for knowledge.

We also note that the central presentation of the face on the screen for each trial, and the presentation cross that preceded it, might have served to train participants to maintain central fixations (see Arizpe, Kravitz, Yovel, & Baker, 2012, for an article on how the start position strongly influences eye fixations within a trial). Millen et al. (2017) presented images randomly to the left or right of a preceding central fixation point. This methodological issue poses a challenge for combined methods approaches to concealed recognition that require minimising eye movements such as cognitive pupillometry (e.g., Lubow & Fein, 1996; Seymour et al., 2013) or ERPs (e.g., Meijer et al., 2007). Considering these points, it is remarkable that, despite few fixations across trials, there were significantly more fixations to the eyes during recognition of familiar faces, a lower proportion of fixations to the inner regions of the face, and distinctly longer fixation durations, thereby signalling recognition despite explicit denial of knowledge and deliberate attempts to conceal recognition.

Our novel eye movement CIT findings demonstrate that: the eyes of familiar faces capture attention during attempts to conceal recognition; key internal face features are attended to differently during familiar and unfamiliar face processing; these attentional differences during familiar face recognition can be used to detect concealed knowledge; and longer fixation durations are a stable marker of recognition across spontaneous and planned countermeasures to deceive. Our findings are consistent with models of face perception research which emphasise the importance of the eyes and inner face recognition in face recognition (Abudarham, Shkiller, & Yovel, 2019; Abudarham & Yovel, 2016). Our new findings during a sequential CIT demonstrate the robustness of fixation duration as a key marker of recognition for detection of concealed face recognition.

We acknowledge, however, that a minority of our participants did not exhibit longer fixation durations during recognition compared to correct rejection of genuinely unknown faces (~ 30%). It is unclear what factors underlie these individual differences in the current experiment. Despite trying to carefully screen participants to determine those who had known lecturers for at least one semester, it is possible that some participants were more familiar with the faces of some lecturers than others. Not all completed familiarity ratings were returned to the experimenter and so the full data set was not available for further analyses. It is also possible that some of our unfamiliar irrelevant faces looked too similar to the familiar probe face or, indeed, to someone else they knew. The important issue of detecting recognition of closely matched or similar faces was neatly outlined in a recent study by Georgiadou et al. (2019), who found that detection of recognition by slower reaction times was improved by matching probe faces to unfamiliar irrelevant faces less closely. We suggest that future research should record familiarity or similarity-to-probe ratings for all faces at the end of the test to further assess the importance of this factor Nonetheless, real-world versions of the test are unlikely to use dissimilar identities since this would violate requirements for fair line-ups. In sum, we conclude that eye movement CITs shows promise for potential field use but that future research should carefully explore factors that contribute to individual variability in fixations as markers of recognition during concealed knowledge.

## Additional file

Additional file 1: **Figure S1.** Heat maps of participants’ fixation data during the countermeasures condition show fixation patterns dispersed across the forehead, ears, eyes, nose, mouth and chin as instructed, both during concealed recognition of familiar probe faces (top right) and honest responses to genuinely unfamiliar faces (bottom right). **Table S1.** Percentage of participants showing a *z*-score consistent with predictions for each measure by condition (DOCX 544 kb)

## Data Availability

The datasets generated and/or analysed during the current study are available in the OSF repository (https://osf.io/k2aut/).

## Footnotes

1 The system built by Chi Ho Chan at University of Surrey uses a four-layer convolutional neural net trained with a CASIA-Webface image set. Faces are matched using the cosine similarity score of the output feature vector.

2 For three of the measures, one participant had the same score for every irrelevant item, resulting in a standard deviation of zero. For these three cases, the z scores were computed using the sd of the whole set of responses for that participant. Note that this is conservative since, if the response to probe items does differ from that of the irrelevant items, the sd of the whole set of responses will always be bigger than the sd of the irrelevant items alone, resulting in lower z scores. In one case, a participant scored the same for every item – they looked at the internal features of every face 100% of the time. In this case, the z scores for probes and irrelevant items were set to zero, since there is nothing to distinguish them.
