# Supplementary material for: Eye see through you! Eye tracking unmasks concealed face recognition despite countermeasures
Source: Cogn Res Princ Implic. 2019 Aug 7;4:23. doi: 10.1186/s41235-019-0169-0 (PMC6684707; doi:10.1186/s41235-019-0169-0)
Supplement: Supplementary file 1 — Figure S1. Heat maps of participants’ fixation data during the countermeasures condition show fixation patterns dispersed across the forehead, ears, eyes, nose, mouth and chin as instructed, both during concealed recognition of familiar probe faces (top right) and honest responses to genuinely unfamiliar faces (bottom right). Table S1. Percentage of participants showing a z-score consistent with predictions for each measure by condition (DOCX 544 kb) [file 41235_2019_169_MOESM1_ESM.docx]

Additional file 1


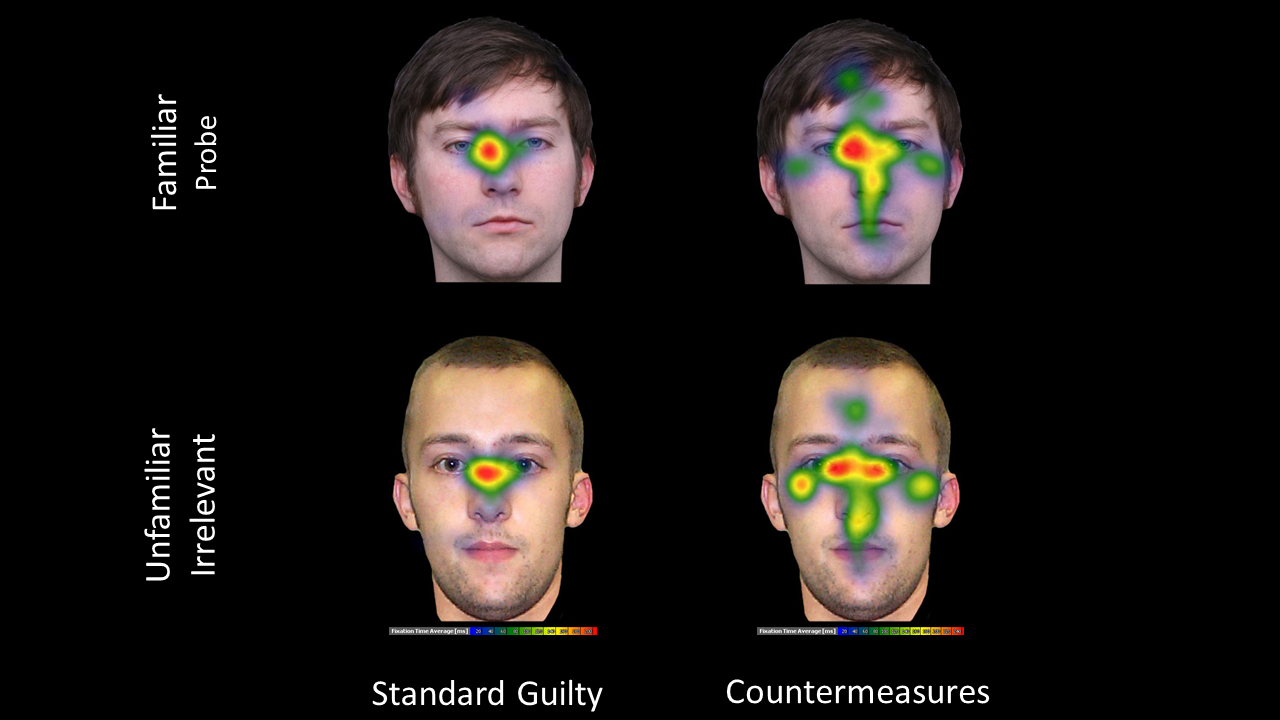


Figure S1. Heat maps of participants’ fixation data during the countermeasures condition show fixations patterns dispersed across the forehead, ears, eyes, nose, mouth and chin as instructed, both during concealed recognition of familiar probe faces (top right) and honest responses the genuinely unfamiliar faces (bottom right).

Table S1. Percentage of participants showing a z-score consistent with predictions for each measure by condition.

|  | Standard Guilty | Countermeasures |
| --- | --- | --- |
| Num. Fix | 30.9 | 46.2 |
| IAs Visited | 43 | 77.5 |
| Prop. Inner | 57 | 83.6 |
| AFD | 68.8 | 76.1 |
| 1st AFD | 66.7 | 67.5 |
| Eyes | 88.5 | 48.9 |
